# A Novel Domain‐Confined Growth Strategy for In Situ Controllable Fabrication of Individual Hollow Nanostructures

**DOI:** 10.1002/advs.201700213

**Published:** 2018-02-26

**Authors:** Luping Tang, Longbing He, Lei Zhang, Kaihao Yu, Tao Xu, Qiubo Zhang, Hui Dong, Chao Zhu, Litao Sun

**Affiliations:** ^1^ SEU‐FEI Nano‐Pico Center Key Lab of MEMS of Ministry of Education Southeast University Nanjing 210096 China; ^2^ Southeast University‐Monash University Joint Research Institute Suzhou 215123 P. R. China

**Keywords:** domain‐confined growth, electron beam, hollow nanocrystals, partially sublimated nanocrystals, single‐particle manipulation

## Abstract

The manipulation and tailoring of the structure and properties of semiconductor nanocrystals (NCs) is significant particularly for the design and fabrication of future nanodevices. Here, a novel domain‐confined growth strategy is reported for controllable fabrication of individual monocrystal hollow NCs (h‐NCs) in situ inside a transmission electron microscope, which enables the atomic scale monitoring of the entire reaction. During the process, the preformed carbon shells serve as nanoreaction cells for the formation of CdSeS h‐NCs. Electron beam (e‐beam) irradiation is demonstrated to be the key activation factor for the solid‐to‐hollow shape transformation. The formation of CdSeS hollow NCs is also found to be sensitive to the volume ratio of the CdSe/CdS NCs to the carbon shell and only those CdSe/CdS NCs with a volume ratio in the range 0.2–0.8 are successfully converted into hollow NCs. The method paves the way to potentially use an e‐beam for the in situ tailoring of individual semiconductor NCs targeted toward future nanodevice applications.

## 1 Introduction

Hollow nanocrystals (h‐NCs) show a unique combination of physical properties including large surface area, low density, and high loading capacity, which enables their application in diverse fields such as micro/nanoreactors,^1^, ^2^ catalysis,^3^ energy storage,^4^, ^5^ drug delivery and biomedicine,^6^ and sensing.^7^ Consequently, the development of synthesis techniques for the controlled design and fabrication of h‐NCs is of great interest. A variety of synthesis methods have been investigated to obtain hollow NCs. These include techniques that adopt a template‐mediated approach,^8^, ^9^ self‐assembly,^10^ galvanic exchange,^11^, ^12^ Kirkendall reaction,^13^ Ostwald ripening,^14^ and surface‐protected etching.^15^ Although these methods have been successful in the synthesis of h‐NCs with different shapes depending upon the reaction and growth in chemical solutions, such liquid phase synthesis strategies currently do not afford fine‐tuning of the resulting structure. Moreover, a detailed understanding of the mechanism of their formation cannot be obtained under these conditions. For example, most of the template‐mediated approaches rely strongly on the quality of the templating material and the method used to etch these templates. Using harsh reaction conditions to remove the core material usually induces unexpected structural collapse of the nanostructure, whereas moderate treatments very often leave residues from the core as impurity.^8^, ^10^ In cases where soft‐templates like liquid interfaces or gas bubbles are used, the assembly of hollow shells around these templates cannot be easily controlled since their sizes, shapes, and uniformity are strongly restricted by the preformed emulsion, vesicle/micelle, or gas bubbles as well as their stability.^16^

In contrast, self‐templating methods, where NCs themselves serve as templates to form h‐NCs, are attractive in view of their relative simplicity.^17^ The growth of an outer shell by consuming the templating NC through ion exchange, Kirkendall effect, Ostwald ripening, or phase transition in solid state can produce clean h‐NCs with improved dimension and shape control.^18^ Great efforts have been made to achieve controlled structure conversion from solid to hollow, for which, electron beam (e‐beam) irradiation has recently been demonstrated to be an efficient tool.^19^ For example, Feng et al. achieved the solid‐to‐hollow transformation of NaYF_4_:Yb,Er NCs through electron beam lithography.^20^ Latham et al. demonstrated that high energy electron irradiation of amorphous metal (Fe, Co, and Ni) nanoparticles eventually converted them into hollow spheres.^21^ Yang et al. showed that ZnO/Al_2_O_3_ core/shell nanowires could be converted into nanotubes by locally etching the ZnO core with an electron beam.^22^ These findings indicate that e‐beam irradiation has definite advantages over other techniques for the fabrication of nanostructures, more specifically, to tailor the structures of individual NCs. This possibility makes this technique a valuable tool for the in situ design and construction of NC‐based nanodevices.

With the minimization trend of optoelectronic devices, researchers have spent much effort to develop multifarious nanomaterials for functional applications. To date, it has already been reported in the literatures that individual semiconductor hollow structures endow with superior properties to apply in nanodevice such as microwave absorber,^23^ single‐photon source,^24^ nanomechanical component,^25^ and so on. However, the highly integrated nanodevice technology is still limited by fine control and tailoring of nanomaterials. In this paper, we report a novel domain‐confined crystal growth strategy for in situ fabrication of individual hollow CdSeS NCs both by thermal treatment and by e‐beam irradiation of CdSe/CdS NC templates. We demonstrate that the sublimation and regrowth of NCs can be tailored by changing the heating temperature and the e‐beam irradiation parameters. In the presence of a preformed thin carbon shell on the templating NCs, the transformation of the partially sublimated CdSe/CdS NCs into hollow CdSeS NCs can be restricted to take place selectively within the shell. Furthermore, it is found that the regrowth of hollow CdSeS NCs is sensitive to the ratio of the volume of the unsublimated CdSe/CdS NC to that of the carbon sphere. It is observed that depending on this ratio, only a certain fraction of the CdSe/CdS NCs can be successfully converted into h‐NC structures.

## 2 Results and Discussion

**Figure 1:**
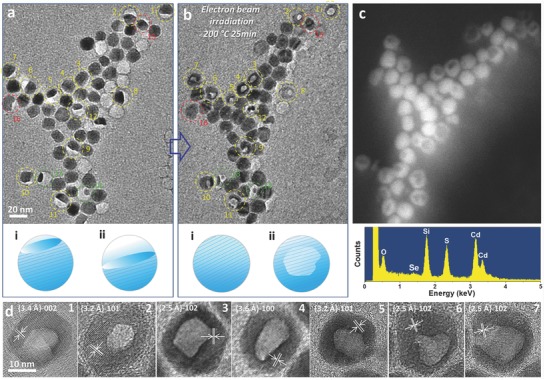
a) TEM image of CdSe/CdS NCs after thermal treatment at 340 °C for 10 min. b,c) TEM image and high‐angle annular dark field (HAADF) scanning transmission electron microscope (STEM) image of the NCs in (a) after irradiation with an electron beam (e‐beam) at 200 °C for 25 min; these images confirm that hollow structures are formed. The corresponding schematic illustrations of two typical shape transformations (i and ii) upon e‐beam irradiation are shown below (a) and (b). Similarly, a representative EDX spectrum from the NCs shown in (c) is included below (c); the signals of Cd, Se, and S can be observed. d) 1–7 show the magnified TEM images of the hollow NCs in (b).

This advanced synthesis procedure^26^ has the advantage that the templating CdSe/CdS NCs have quite a uniform size distribution with an average particle size of around 15.4 nm (Figure S1, Supporting Information). First, several regions of interest are selected and marked by the transmission electron microscope (TEM) stage. Through e‐beam irradiation, the residual surfactant molecules on the CdSe/CdS NC surfaces and the carbohydrate molecules in the TEM chamber are carbonized at the imaging region, and carbon shells are subsequently formed onto the NC surfaces. In this process, the thickness of the carbon shells can be precisely controlled by the e‐beam intensity and the irradiation time (see more details in the Experimental Section). In the experiment here, the thickness of the carbon shells is controlled to be 1–2 nm by monitoring the growth process of the carbon shells. **Figure** **1**a,b shows the formation of h‐NCs by e‐beam irradiation of partially sublimated CdSe/CdS NCs at 200 °C (more information on the prior sublimation of CdSe/CdS NCs at 340 °C is given in Figure S2, Supporting Information). It is seen in Figure 1 that a significant number of shape transformations of the residual CdSe/CdS NCs (as marked by the numbered dotted rings) occur as a result of e‐beam irradiation. Interestingly, most of the half‐moon‐like CdSe/CdS NCs gradually turn into bowl‐like or hollow spheres (Nos. 1–12). Others including empty box‐like forms and NCs suffering either very little sublimation or rather severe sublimation show significantly different evolution patterns. For the empty carbon boxes, no significant growth is observed on their inner shells. On the other hand, NCs having very small (Nos. 13 and 14) or very large residual cores (Nos. 15 and 16) eventually turn into discrete islands or spheres, respectively. These evolutions prove that the e‐beam induced regrowth of the partially sublimated CdSe/CdS NCs is mainly restricted inside the carbon shells. The corresponding scanning transmission electron microscope (STEM) image shown in Figure 1c further confirms that no significant nucleation or Ostwald ripening is observed outside the carbon shells. In addition, the energy‐dispersive X‐ray (EDX) spectral data acquired in STEM mode show that the types of chemical elements of the NCs remain unchanged, indicating that Cd, Se, and S still partially remain after the transformation of the solid CdSe/CdS NCs into hollow CdSeS NCs. It is also noted that the NCs maintain their crystalline structure through the entire transformation process. As displayed in Figure 1d, high‐resolution TEM images of the obtained h‐NCs sequentially marked from 1 to 7 in Figure 1b show clear lattice fringes corresponding to CdSeS (002), (101), (102), and (100) crystal planes. The voids formed inside the h‐NCs are rather irregular, which may be due to inhomogeneous nucleation and anisotropic growth.

**Figure 2:**
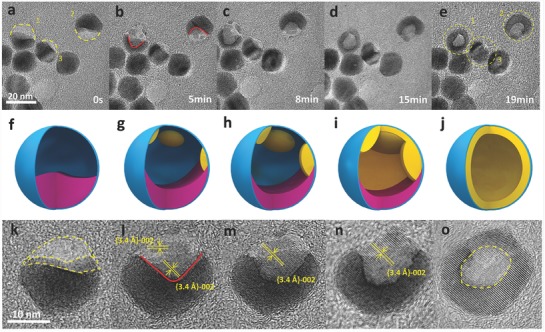
a–e) Captured time‐series video frames illustrating the domain‐confined growth of partially sublimated CdSe/CdS NCs induced by e‐beam irradiation in vacuum at 200 °C; prior to e‐beam, the NCs were heated at 340 °C for 10 min in a TEM equipped with a heating sample holder. Growth schemes and high‐resolution transmission electron microscopy (HRTEM) images of particle 2 indicated in (a) are shown below the corresponding images (a)–(e). f–o) (1) Partially sublimated NCs are formed after annealing at 340 °C for 10 min. (2) Isolated regions where growth of a new phase occurs randomly along the inside surface of the carbon shell, while the rest of the NCs continue to sublimate; the interface between the liquid‐like phase and the gaseous fraction within the carbon shell forms a deep V‐shaped crater [indicated by the red solid line in (l)]. (3) Isolated regions grow larger and other lattice planes are gradually assimilated into the lattice plane denoted by the arrows. (4) The newly added crystal planes grow into a whole larger crystal covering the entire surface with lattice spacing of ≈3.4 Å, which is in‐between CdSe ({002}‐3.5 Å) and CdS ({002}‐3.3 Å); this finding implies that the composition of the new crystal could be CdSeS {002}. (5) The formation of a hollow structure is clearly illustrated.

To further reveal the mechanism of the dynamic structure evolution observed in Figure 1, we monitored the regrowth of several half‐moon‐like CdSe/CdS NCs in situ. The shape transformation of these structures by stepwise surface reconstruction is shown in **Figure** **2**a–e for the NCs marked 1, 2, and 3. Upon continued e‐beam irradiation, the free surfaces of these structures, which are initially nearly flat, turn concave, and finally change to hollow spheres. All the NCs mainly regrow at the expense of themselves and are therefore domain‐confined reconstructions. In comparison, the partially sublimated NCs in regions without e‐beam irradiation (after position tracking) maintain their initial shapes (Figure S4, Supporting Information). This observation proves that the structural regrowth of the NCs is caused mainly by e‐beam irradiation rather than by thermal diffusion. Certainly, thermal heating may also accelerate the process of shape change. Schematics and high‐resolution images in Figure 2f–o describe the regrowth process under e‐beam irradiation for particle 2. TEM images confirm that the NC remains crystalline with a distinct lattice spacing of 3.40 Å, which lies in‐between the lattice spacing values for the (002) surfaces of CdSe (3.52 Å) and CdS (3.34 Å) indicating that the chemical composition of the newly formed shell may be CdSeS. Another interesting feature in the structure evolution is that the shell regrowth is rather an anti‐Ostwald ripening process. As illustrated by the schematic images, multisite nucleation takes place at the early stages of e‐beam irradiation. These nuclei grow into small islands mainly at the expense of the residual NC surface, which undergoes decomposition. The continuous growth and coalescence of the islands as well as the reduction of the residual NC eventually lead to the formation of a hollow NC inside the carbon shell. Since the regrowth is induced by e‐beam, an effective control of the radiation dose can fine tune the formed hollow NC to be well sealed or bowl‐like.

**Figure 3:**
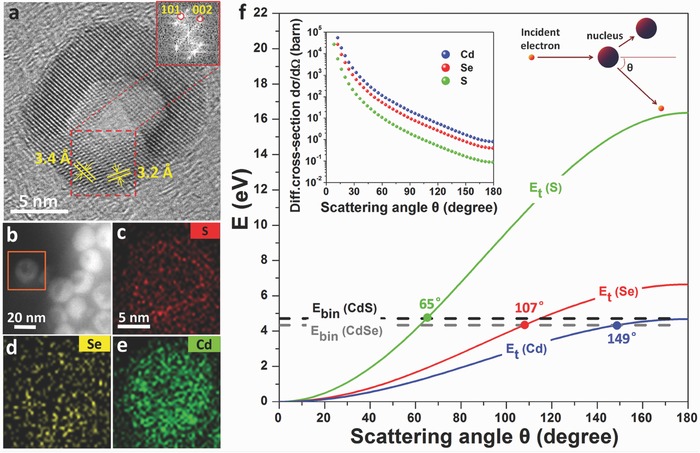
a) Representative HRTEM image of a hollow structure showing lattice fringes with spacing corresponding to CdSeS ({002}‐3.4 Å and {101}‐3.2 Å). Fourier transform of the image in the area enclosed by red dashed lines is shown in the inset. b) HAADF‐STEM image of hollow NCs. c–e) S, Se, and Cd elemental maps obtained from EDX mapping indicating that the elements are almost evenly distributed in the hollow NCs. f) Energy transfer efficiency (*E*_t_) from 200 keV electrons to Cd (blue), Se (red), and S (green) as a function of the scattering angle θ. Horizontal dashed lines indicate the limit beyond which, the transferred energy is greater than the binding energy of CdSe (gray) and CdS (black) and is sufficient to induce the sublimation of atoms.

It has been known that the morphology of CdSe or CdS NCs grown in oleic acid with octadecene surfactant can be effectively regulated by adjusting the reaction temperature.^27^ In this case, the geometry of the resulting NCs is controlled by ligand‐mediated growth kinetics and surface energy minimization.^28^ In sharp contrast, the observed structural evolution of the NCs in our experiment is quite different from that prevalent in solutions. This gas‐phase regrowth under e‐beam irradiation is absolutely ligand free and the formation of a void inside the NC also increases the free surface area of the NC. Since the surface energies of the carbon shell and CdSe/CdS are 54–125^29^ and 280–900 mJ m^−2^,^30^, ^31^ respectively, the total surface energy of the whole system including the carbon shell increases due to the increase in free surface in the NC. In other words, excitation from an external source appears to be a prerequisite to transform the partially sublimated NCs into hollow structures. One possible mechanism of this structure evolution is based on the fact that Cd, Se, and S atoms can be peeled off from the CdSe/CdS surface by the impinging high energy electrons and redeposited elsewhere. Such dynamic decomposition and recombination processes enable the redistribution of the NC mass and can lead to the formation of a hollow shell. As shown in **Figure** **3**a and Figure 2l–o, a distinguishing characteristic of the formed hollow NC is its improved crystallinity following the e‐beam treatment. The ring‐like hollow NC tends to convert into a monocrystalline structure. EDX mappings in Figure 3b–e show that the hollow NC comprises all the three elements distributed evenly, indicating that the as‐synthesized core–shell structure of the CdSe/CdS NC is finally transformed into stoichiometric CdSeS (the EDX spectrum is shown in Figure S5, Supporting Information).

During e‐beam excitation, beam‐induced heating and sputtering are the two major effects, which cause the decomposition and regrowth of NCs. To estimate the sputtering effect, a model based on electron scattering is used to calculate the transferred energy *E*_t_ through electron‐atom collisions, which is given by^32^, ^33^

(1) $$E_{t}=\frac{2E_{0}\left(E_{0}+2mc^{2}\right)}{Mc^{2}}\cdot {\left(\sin\frac{\theta }{2}\right)}^{2}$$

where *M*, *m*, *c*, *E*_0_, and θ are the values of atomic mass, electron mass, speed of light in vacuum, electron energy, and scattering angle, respectively. Substituting the corresponding parameters for Cd, Se, and S, the relationship of *E*_t_ as a function of θ is calculated and plotted in Figure 3f. These results indicate that *E*_t_ increases with scattering angle. At θ > 90° or θ = 180°, the backscattering of electrons is inevitable. As a result, *E*_t_ has distinct effects on atoms, although at small angles, electrons scatter with a negligible *E*_t_ value of < 1eV. The binding energies of CdS and CdSe (4.71 and 4.34 eV, respectively), represent the energy required to remove an atom from the upper surface layer of the material into vacuum during ion sputtering.^34^ The surface atoms on the partially sublimated CdSe/CdS can be ejected upon e‐beam irradiation because, the transferred energy *E*_t_ of S, Se, and Cd can exceed the respective atom binding energies when the electron deflected angles are larger than 65°, 107°, and 149°, respectively (the point of intersection of the dashed horizontal line and the solid line in Figure 3f). This observation is consistent with results described in previous studies.^35^ During the irradiation, the atoms of the newly formed CdSeS can also be peeled off to some extent by the impinging high energy electrons and redeposited on the inner surface of the carbon shell to form a hollow structure reaching an equilibrium. Moreover, the displacement rate *P* of each atom is determined by the equation *P* = σ_tot_ · *j*, where σ_tot_ is the total displacement cross‐section and *j* is the beam current density. The total displacement cross‐section is obtained by integrating the differential cross‐section dσ/dΩ and gives the probability for an atom to be recoiled into the solid angle dΩ^36^ The expression for dσ/dΩ is as follows^37^

(2) $$\begin{array}{l} \frac{d\sigma }{d\Omega }\;=\;\frac{Z^{2}r_{c1}^{2}}{4}\cdot \frac{\left(1-\beta^{2}\right)}{\beta^{4}}\cdot \csc^{4}\left(\frac{\theta }{2}\right)\cdot \\ \;\;\;\;\left[1\;-\;\beta^{2}\sin^{2}\frac{\theta }{2}\;+\;\pi \alpha \beta \sin\frac{\theta }{2}\left(1\;-\;\sin\;\frac{\theta }{2}\right)\right] \end{array}$$

Here $\alpha \;=\;\frac{Ze^{2}}{\hbar c}\;=\;\frac{Z}{137}$, $\beta =\;\frac{\nu }{c}\;=\;\sqrt{1\;-\;{\left(1{+}^{eV}/m_{0}c^{2}\right)}^{-2}}$, *r*_*c*1_ is the classical electron radius, and *Z* is the atomic number. The illustration on the top left of Figure 3f shows the differential cross‐section dσ/dΩ as a function of the scattering angle θ. The value of dσ/dΩ decreases rapidly with the increasing θ and is minimum for a central impact θ = 180°. While the differential cross‐section is always higher for small energy transfer, it can be seen that dσ/dΩ increases with increasing *Z*, which means that heavier atoms should be displaced more easily (in this work, Cd>Se>S). In most of the half‐moon‐like CdSe/CdS NCs, the sputtering atoms were deposited randomly on the inner surface of the carbon shell, which served as a template. Some isolated islands can be formed initially, which may grow when atomic concentrations are increased. Small islands can further combine with large islands through Ostwald‐ripening process when they come in contact with each other. Consequently, a new layer is formed, which covers the whole hollow structure. Moreover, according to ref. 38 and 39, besides e‐beam sputter‐redeposition, the extremely fast thermally induced surface diffusion and wetting behavior of the material may also accelerate the process of shape change. In addition to sputtered atoms, atoms outside the shell may also deposit inside the carbon shell, which in turn, can form new shells. This could explain the increased atomic proportion of Se (Figure 3d). Thus the volume of the hollow space in the newly formed structures is nearly equivalent to or slightly smaller than the volume of the void in the initial partially sublimated NCs. Nevertheless, in NCs with very little sublimation, due to the narrow available space for the formation of isolated islands, crystal growth depends mainly on the reconstruction of the outer atoms along the original interface in the carbon shell (Figure S6, Supporting Information).

**Figure 4:**
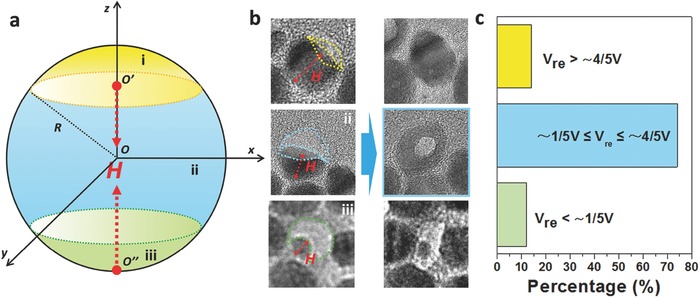
a) Model used to calculate the residual volume (*V*_re_) of the partially sublimated CdSe/CdS NC. *V*_re_ can be estimated from the height marked by *H* using the spherical cap model (i.e., $V_{\mathrm{re}}=\left(\frac{3H^{2}}{4R^{2}}\;-\;\frac{H^{3}}{4R^{3}}\right)\times V$). b) TEM images of the typical three types of shape evolution. Type‐i is where there is no significant void in the center of the NC after e‐beam irradiation. Type‐ii is where the formation of h‐NC after e‐beam irradiation is clearly seen. Type‐iii is where the residual NC maintains the island shape. Statistical calculation of a number of NCs indicates that the *H*/*R* values for type‐i, type‐ii, and type‐iii are around 1.4–2, 0.6–1.4, and 0–0.6, respectively. Accordingly, the residual CdSe/CdS NCs are divided into three sorts depending on the value of *V*_re_, i.e., *V_re_* > ≈ 4/5V (yellow), $\approx \;1/5V\;\leq \;V_{\mathrm{re}}\leq \;\approx \;\mathrm{4/5V}$ (blue), and *V*_re_ < ≈ 1/5V(green). c) Percentage yield of CdSeS h‐NCs formation starting from treated CdSe/CdS NCs.

Statistical calculations have shown that almost all of those CdSe/CdS NCs with a volume ratio in the range 0.2–0.8 can be successfully converted into hollow NCs under irradiation with a proper beam intensity, which portended high repeatability for solid‐to‐hollow single‐particle manipulation. **Figure** **4**a shows a schematic of the estimation of the residual volume of the partially sublimated NCs (details are shown in the section on “The residual volume calculation and classification method” in the Supporting Information). The letter *H* denotes the height of the residual CdSe/CdS NC after partial sublimation. The final structures of the end products can be classified into three types depending on the value of *H*/*R* (i.e., the residual volume) in the partially sublimated NCs, shown in Figure 4b. (1) Solid quasi‐spheres (*V*_re_ > ≈ 4/5V), (2) hollow structures ($\approx 1/5V\;\leq \;V_{\mathrm{re}}\leq \;\approx \mathrm{4/}5V$), and (3) remaining unchanged with respect to the initial structure (*V*_re_ < ≈ 1/5V). Thus, it is clear that by controlling the residual volume of the NCs, solid‐to‐hollow single‐particle manipulation can be achieved. Figure 4c further reveals that the formation of CdSeS hollow NCs is also found to be sensitive to the volume ratio of the CdSe/CdS NCs to the carbon shell and only those CdSe/CdS NCs with a volume ratio in the range 0.2–0.8 are successfully converted into hollow NCs. It is noted here that only partially sublimated CdSe/CdS NCs have been considered for the statistical calculation.

## 3 Conclusion

In summary, a novel domain‐confined growth strategy for controllable fabrication of individual hollow CdSeS NCs has been achieved through an in situ e‐beam activation process in TEM. The recrystallization of the h‐NCs is exclusively confined to inside the carbon shells. The dominant role of electron beam in the formation of CdSeS h‐NCs is demonstrated. Moreover, the volume ratio of the CdSe/CdS NC to the carbon shell is the key factor for the formation of hollow CdSeS NCs. Three types of shape evolution can be observed; only CdSe/CdS NCs with a volume ratio of around 0.2–0.8 can successfully yield CdSeS h‐NCs. Anti‐Ostwald ripening is observed in the formation process of CdSeS h‐NCs and the mechanism of surface energy increase is discussed and found to be related to the e‐beam effect. Our findings here provide valuable knowledge on the interactions between a high‐energy electron beam and semiconductor nanocrystals, as well as a way for better understanding the formation mechanism of hollow nanostructures. With the improvement of preparation technology, such as the material promotion, production cost, and repeatability, the practical application of these individual hollow nanostructures would become more and more extensive.

## 4 Experimental Section

*Preparation of Templating CdSe/CdS NCs*: CdSe/CdS core/shell solid NCs were synthesized by epitaxial growth of ≈16 monolayers of CdS shells on the CdSe crystal seeds using the successive ionic layer adsorption and reaction method. The obtained NCs were then washed and purified by precipitating them two to three times with ethanol and redispersing in hexane. More details on this procedure can be found in ref. 25.

*Structural Characterization*: The NC structures were characterized and analyzed by both X‐ray diffraction (Rigaku D/max 2500 VL/PC diffractometer) and transmission electron microscopy (TEM; Tecnai G20 and Titan 80‐300 from field electron and ion (FEI) company). Normal imaging was carried out under a low e‐beam density to avoid beam‐induced effects. Typically, the beam density for Tecnai G20 was 30 A cm^−2^ while that for Titan 80–300 was 60 A cm^−2^. Dark field imaging and EDX spectroscopy were performed with Titan 80‐300 in STEM mode. The EDX mapping was carried out with a step of 0.75 nm per point and a dwell time of 500 ms per point.

*Thermal Treatments and Electron Beam Irradiation*: For the thermal treatment, a TEM heating holder (Aduro) was used from Protochips company. Heat was generated by an electrical heating chip (E‐chip). The E‐chip had a supporting silicon nitride film with arrays of holes that served as observation windows and the nominal ramp rate of the heating e‐chip was 1000 °C ms^−1^. More details on the chip configuration can be found in ref. 29. In a typical experiment, a small drop of purified CdSe/CdS NC solution was dispersed onto the E‐chip with a micropipette. After drying, the E‐chip was mounted onto the holder, which was introduced into the TEM stage for thermal heating.

To enable sublimation of the CdSe/CdS NCs, a heating temperature of 340 °C was fixed. The sublimation process was controlled by monitoring the shape evolution of the NCs in situ. Once the NC volume decreased and reached target values, sublimation was stopped by resetting the heating temperature to room temperature. For the subsequent thermal annealing and regrowth of CdSeS shells, the heating temperature was set to 200 °C.

For e‐beam irradiation, a much more intense beam intensity was applied as compared to normal imaging. In order to carbonize the surfactants into carbon shells, a beam intensity of 30–60 A cm^−2^ was selected. Typically, the regions of interest were first selected and marked, and then each of them was irradiated under the e‐beam for 3–5 min. The thickness and crystallinity of the formed carbon shell were controlled by real‐time monitoring of the carbon deposition and growth.^40^ For e‐beam induced regrowth of partially sublimated CdSe/CdS NCs, the beam intensity was maintained in the range 60–90 A cm^−2^. The irradiated regions were marked and tracked so as to identify the effects of irradiation by comparing these with regions without irradiation.

## Conflict of Interest

The authors declare no conflict of interest.

## Supporting information

Supplementary

## References

[1] J. Gao , X. Zhang , Y. Lu , S. Liu , J. Liu , Chem. Eur. J. 2015, 21, 7403. 25821159 10.1002/chem.201500532

[2] S. M. Kim , M. Jeon , K. W. Kim , J. Park , I. S. Lee , J. Am. Chem. Soc. 2013, 135, 15714. 24102162 10.1021/ja4083792

[3] X. Xu , Z. Zhang , X. Wang , Adv. Mater. 2015, 27, 5365. 26172949 10.1002/adma.201500789

[4] H. Liu , W. Li , D. Shen , D. Zhao , G. Wang , J. Am. Chem. Soc. 2015, 137, 13161. 26414170 10.1021/jacs.5b08743

[5] H. Ren , R. Yu , J. Wang , Q. Jin , M. Yang , D. Mao , D. Kisailus , H. Zhao , D. Wang , Nano Lett. 2014, 14, 6679. 25317725 10.1021/nl503378a

[6] Y. Zhang , B. Y. W. Hsu , C. Ren , X. Li , J. Wang , Chem. Soc. Rev. 2015, 44, 315. 25310644 10.1039/c4cs00199k

[7] L. Wang , Z. Lou , T. Fei , T. Zhang , J. Mater. Chem. 2011, 21, 19331.

[8] G. Chen , S. Desinan , R. Rosei , F. Rosei , D. Ma , Chem. Commun. 2012, 48, 8009. 10.1039/c2cc33396a 22773309

[9] P. P. Yang , Y. Xu , L. Chen , X. C. Wang , Q. Zhang , Langmuir 2015, 31, 11701. 26434608 10.1021/acs.langmuir.5b03192

[10] X. L. Fang , X. J. Zhao , W. J. Fang , C. Chen , N. F. Zheng , Nanoscale 2013, 5, 2205. 23400270 10.1039/c3nr34006f

[11] Y. J. Xiong , B. Wiley , J. Y. Chen , Z. Y. Li , Y. D. Yin , Y. N. Xia , Angew. Chem., Int. Ed. 2005, 44, 7913. 10.1002/anie.200502722 16304650

[12] E. Gonzalez , J. Arbiol , V. F. Puntes , Science 2011, 334, 1377. 22158813 10.1126/science.1212822

[13] H. J. Fan , U. Gösele , M. Zacharias , Small 2007, 3, 1660. 17890644 10.1002/smll.200700382

[14] X. Wang , H. Fu , A. Peng , T. Zhai , Y. Ma , F. Yuan , J. Yao , Adv. Mater. 2009, 21, 1636.

[15] Y. J. Wong , L. Zhu , W. S. Teo , Y. W. Tan , Y. Yang , C. Wang , H. Chen , J. Am. Chem. Soc. 2011, 133, 11422. 21732677 10.1021/ja203316q

[16] X. Fan , Z. Zhang , G. Li , N. Rowson , Chem. Eng. Sci. 2004, 59, 2639.

[17] Q. Zhang , W. Wang , J. Goebl , Y. Yin , Nano Today 2009, 4, 494.

[18] X. Wang , J. Feng , Y. Bai , Q. Zhang , Y. Yin , Chem. Rev. 2016, 116, 10983. 27156483 10.1021/acs.chemrev.5b00731

[19] A. E. Mel , C. Bittencourt , Nanoscale 2016, 8, 10876. 27172892 10.1039/c6nr02293f

[20] W. Feng , L. D. Sun , Y. W. Zhang , C. H. Yan , Small 2009, 5, 2057. 19507152 10.1002/smll.200900404

[21] A. H. Latham , M. E. Williams , Langmuir 2008, 24, 14195. 19360944 10.1021/la7035423

[22] Y. Yang , R. Scholz , A. Berger , D. S. Kim , M. Knez , D. Hesse , U. Gösele , M. Zacharias , Small 2008, 4, 2112. 18989863 10.1002/smll.200800795

[23] M. Cao , H. Lian , C. Hu , Nanoscale 2010, 2, 2619. 20967389 10.1039/c0nr00674b

[24] J. Geng , B. Liu , L. Xu , F. N. Hu , J. J. Zhu , Langmuir 2007, 23, 10286. 17718525 10.1021/la701299w

[25] Z. W. Shan , G. Adesso , A. Cabot , M. P. Sherburne , S. A. S. Asif , O. L. Warren , D. C. Chrzan , A. M. Minor , A. P. Alivisatos , Nat. Mater. 2008, 7, 947. 18931673 10.1038/nmat2295

[26] Y. Ghosh , B. D. Mangum , J. L. Casson , D. J. Williams , H. Htoon , J. A. Hollingsworth , J. Am. Chem. Soc. 2012, 134, 9634. 22578279 10.1021/ja212032q

[27] J. J. Li , Y. A. Wang , W. Guo , J. C. Keay , T. D. Mishima , M. B. Johnson , X. Peng , J. Am. Chem. Soc. 2003, 125, 12567. 14531702 10.1021/ja0363563

[28] B. Lim , H. Kobayashi , P. H. C. Camargo , L. F. Allard , J. Liu , Y. Xia , Nano Res. 2010, 3, 180.

[29] R. Lian , H. Yu , L. He , L. Zhang , Y. Zhou , X. Bu , T. Xu , L. Sun , Carbon 2016, 101, 368.

[30] L. Liu , Z. Zhuang , T. Xie , Y. Wang , J. Li , Q. Peng , Y. Li , J. Am. Chem. Soc. 2009, 131, 16423. 19902978 10.1021/ja903633d

[31] A. S. Barnard , H. Xu , J. Phys. Chem. C 2007, 111, 18112.

[32] R. F. Egerton , P. Li , M. Malac , Micron 2004, 35, 399. 15120123 10.1016/j.micron.2004.02.003

[33] R. F. Egerton , R. McLeod , F. Wang , M. Malac , Ultramicroscopy 2010, 110, 991.

[34] J. M. Matxain , J. M. Mercero , J. E. Fowler , J. M. Ugalde , J. Phys. Chem. A 2004, 108, 10502.

[35] Y. Liu , Y. Sun , Nanoscale 2015, 7, 13687. 26213998 10.1039/c5nr03523f

[36] F. Banhart , Rep. Prog. Phys. 1999, 62, 1181.

[37] C. R. Bradley , Tech. Rep. Arch. Image Libr. 1988, 6, 4405.

[38] K. Y. Niu , J. Park , H. M. Zheng , A. P. Alivisatos , Nano Lett. 2013, 13, 5715. 24131312 10.1021/nl4035362

[39] A. E. Mel , M. L. Leopoldo , B. Marie , P. Y. Tessier , D. Ke , C. H. Choi , H. J. Kleebe , K. Stephanos , B. Carla , S. Rony , ACS Nano 2014, 8, 1854.24476494

[40] L. He , T. Xu , J. Sun , K. Yin , X. Xie , L. Ding , H. Xiu , L. Sun , Carbon 2012, 50, 2845.

