# Supplementary material for: A Novel Domain‐Confined Growth Strategy for In Situ Controllable Fabrication of Individual Hollow Nanostructures
Source: Adv Sci (Weinh). 2018 Feb 26;5(5):1700213. doi: 10.1002/advs.201700213 (PMC5979780; doi:10.1002/advs.201700213)
Supplement: Supplementary file 1 — Supplementary [file ADVS-5-1700213-s001.pdf]

Copyright WILEY-VCH Verlag GmbH & Co. KGaA, 69469 Weinheim, Germany, 2018.

Supporting Information

for *Adv. Sci.*, DOI: 10.1002/advs.201700213

A Novel Domain-Confined Growth Strategy for In Situ Controllable Fabrication of Individual Hollow Nanostructures

Luping Tang, Longbing He, Lei Zhang, Kaihao Yu, Tao Xu, Qiubo Zhang, Hui Dong, Chao Zhu, and Litao Sun**

Copyright WILEY-VCH Verlag GmbH & Co. KGaA, 69469 Weinheim, Germany, 2016.

Supporting Information

A novel domain-confined growth strategy for in-situ controllable fabrication of individual hollow nanostructures

Luping Tang, Longbing He, Lei Zhang, Kaihao Yu, Tao Xu, Qiubo Zhang, Hui Dong, Chao Zhu and Litao Sun**

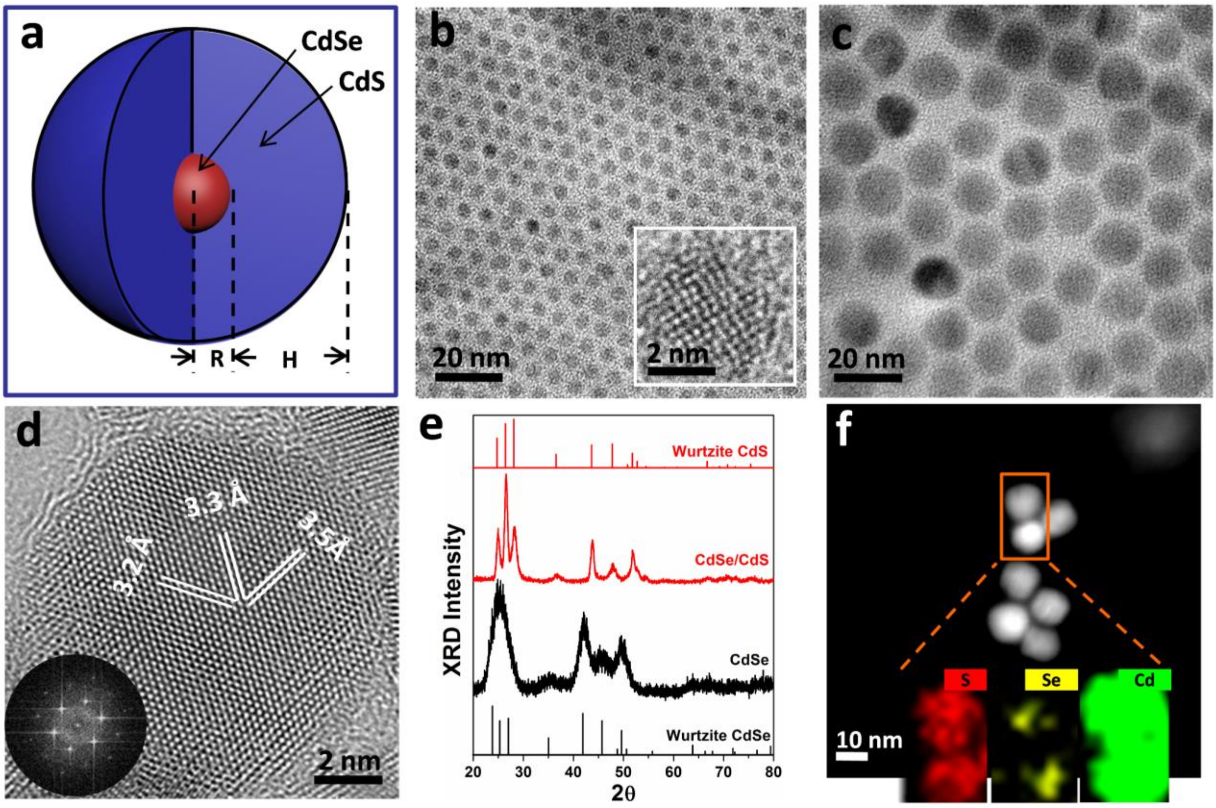

Figure S1. a) A schematic of the NC structure, where R is the radius of the CdSe core and H is the CdS shell thickness. b) A transmission electron microscopy image (TEM) of the CdSe core. Inset shows a typical high-resolution TEM (HRTEM) image. c) TEM image of CdSe/CdS core/shell NCs with R=2.0 nm and H=5.7 nm. d) A representative HRTEM image of spherical CdSe/CdS NCs; inset shows the Fourier transform of the image. e) XRD patterns of the as-synthesized CdSe cores and CdSe/CdS NCs. Vertical lines represent standard Wurtzite CdSe and CdS bulk reflections. f) High angle annular dark field (HAADF) scanning transmission electron microscope (STEM) image of CdSe/CdS NCs in their initial state at room temperature and the corresponding S, Se and Cd elemental maps obtained by EDX mapping.

Sublimation of CdSe/CdS NCs:

Figure S2 shows the sublimation process of CdSe/CdS NCs heated at 340 °C. Thin carbon shells are clearly observed with shapes that are identical to the initial CdSe/CdS NCs. Due to the confinement of the carbon shells, the rate of sublimation of the core in the CdSe/CdS NCs is much more moderate. The shape evolution of the CdSe/CdS NC is significantly influenced by the presence of the carbon shell. As shown in Figure S2h for the whole sublimation process of two typical NCs (marked by dotted circles in Figure S2a), the ablation of the CdSe/CdS routinely occurs from one end of the shell to the other. The surfaces also vary from nearly flat to convex, possibly governed by surface energy considerations.[1] This shell-modulated sublimation routine obviously differs from the case of bare NCs where sublimation directly leads to volume shrinkage of the NCs. Noticeably, the CdSe/CdS NCs maintain their crystalline structure during sublimation (Figure S3, Supporting Information). Moreover, no significant deformation and shell fractures are observed on the carbon shells indicating that these may be quite rigid.

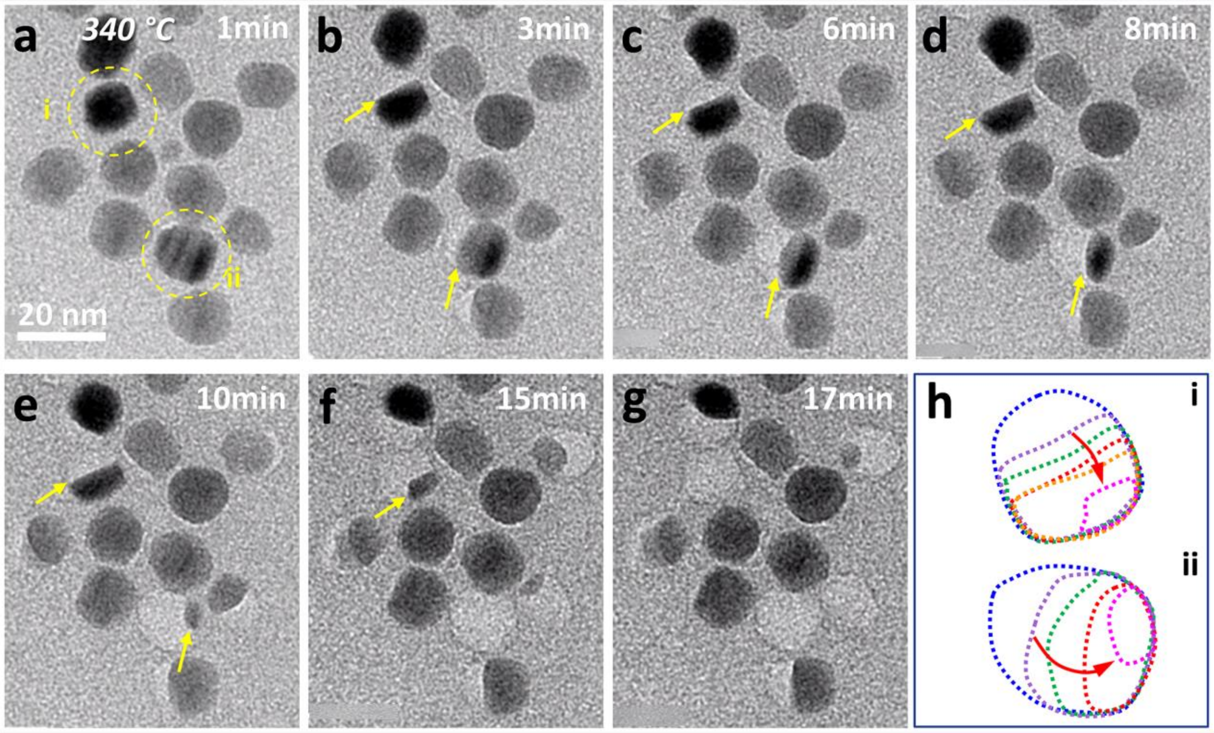

Figure S2. a-g) Sequence of bright-field TEM images during an isothermal experiment at 340°C showing the sublimation of CdSe/CdS NCs. h) Monitored shape contours of the CdSe/CdS NC (obtained from the TEM images in a-g).

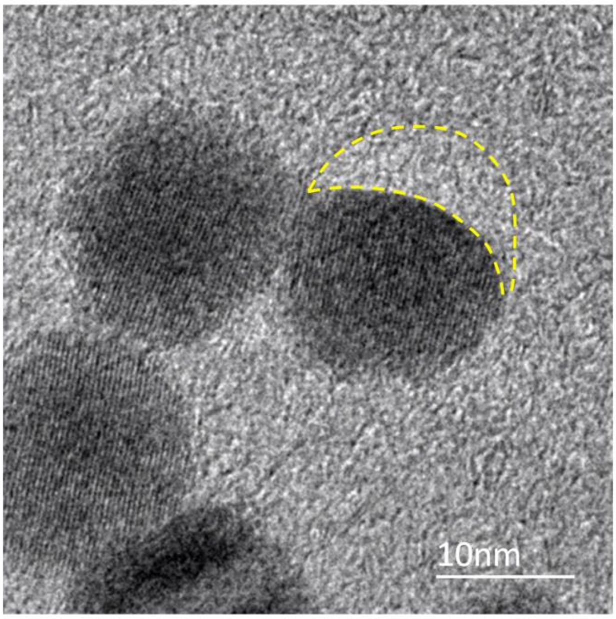

Figure S3. High-resolution TEM image of CdSe/CdS NCs during heat treatment at 340 °C. The lattice fringes indicate that the NC remains solid and crystalline during sublimation.

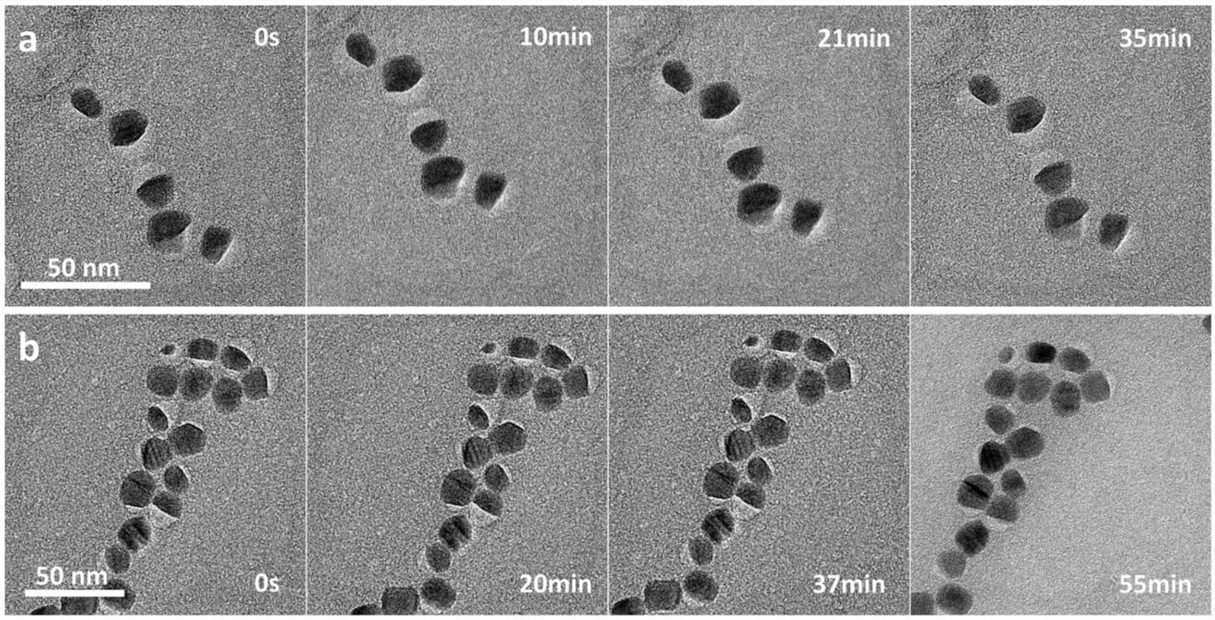

Figure S4. TEM images showing shape evolutions in two random groups of partially sublimated CdSe/CdS NCs (after annealing at 340℃ for 10 min; see section on Methods for more details) at 200℃ for different heating times (35 and 55 min); the electron beam illuminated the samples only at the moment of taking pictures.

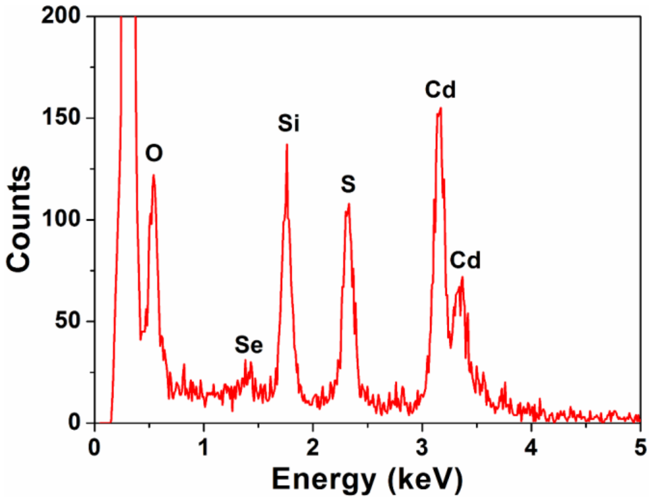

Figure S5. The integrated EDX spectrum corresponding to the drift corrected element mapping in Figure 3c-e indicating that the ratio of the amount of the Se and S is nearly 0.323, which is similar to that of another fifteen hollow NCs selected randomly.[2]

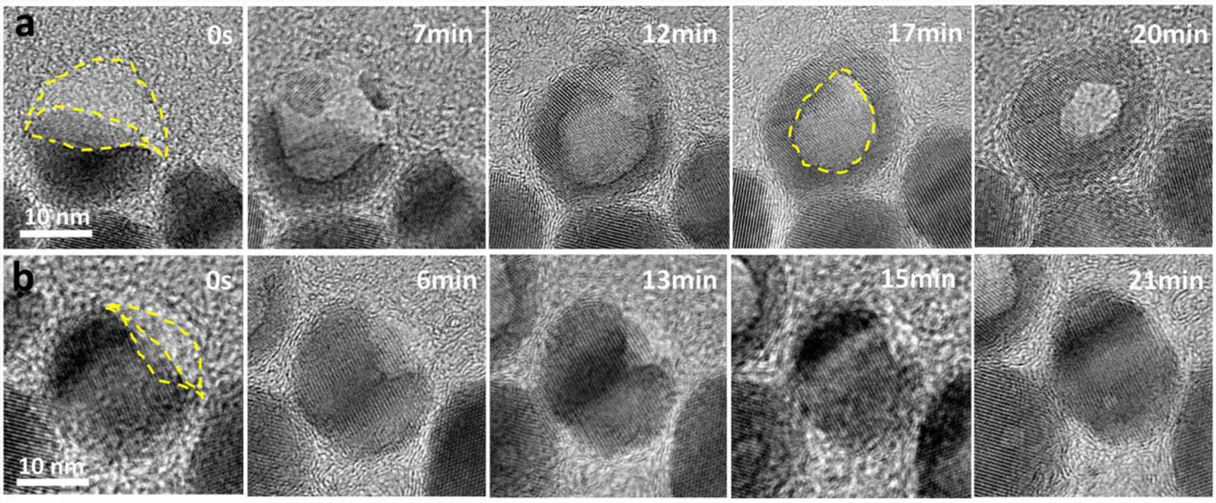

Figure S6. Sequential HRTEM images recorded in situ to monitor the dynamic regeneration of particle 1 ($\sim 1/5V \leq V_{re} \leq \sim 4/5V$) and 3 ($V_{re} > \sim 4/5V$) in Figure 3a. The final structure depends on the residual volume of the partially sublimated NCs before electron beam irradiation. These structures transform at the end of the process into: (a) hollow structures, and (b) solid quasi-spheres. These observations indicate that the availability of sufficient space in the carbon shell is a pre requisite for the formation of hollow structures.

The residual volume calculation and classification method:

From the TEM image of CdSe/CdS NCs showed in Figure S1c, it can be deduced that the NCs are quasi-spherical with uniform size. We have therefore approximated them to be standard spheres in our calculation of the volume (*V*) of NCs. In addition, the interface between the gas phase and the liquid-like phase inside the carbon shell is assumed to be planar to simplify both the calculation and classification of the residual volume of the partially sublimated CdSe/CdS NCs. Besides, taking into consideration the protective nature of the carbon shell, it is assumed that the morphology of the NCs remain unchanged after partial sublimation at 340 ℃. Under these conditions, the residual volume (V_{re}) of the partially sublimated NCs is given by the following equation (the schematic diagram is given in Figure 4a):

$$V_{re} = \left(\frac{3H^2}{4R^2} - \frac{H^3}{4R^3} \right) \times V$$

where R is the radius of sphere, H is the height of the residual CdSe/CdS NC after partial sublimation corresponding to $O'O''$ in the schematic diagram marked with a red dotted line (Figure 4a and 4b (left)). The H values of the NCs No.1–16 in Figure 1a are 9.84, 9.83, 10.48, 9.78, 6.5, 10.32, 10.38, 9.18, 8.36, 10.46, 8.14, 6.32, 3.82, 2.58, 13.39, 14.23 nm, respectively. The partially sublimated NCs can be broadly divided into three types based on the H/R value : 1.4–2, 0.6–1.4 and 0–0.6. (R=15.4 nm). Correspondingly, the volume can be divided into three intervals: $V_{re} > \sim 4/5V$, $\sim 1/5V \le V_{re} \le\sim 4/5V$, and $V_{re} < \sim 1/5V$; this classification is both useful and convenient to describe the experimental observations.

Energy transfer from electron beam to atoms:

The transferred energy (E_t, in eV) is determined by the following equation:[3,4]

$$E_t = E_{\max}\left(\sin\frac{\theta}{2}\right)^2$$

$$E_{\max} = \frac{2ME_0\left(E_0 + 2mc^2\right)}{\left(m+M\right)^2 c^2 + 2ME_0}$$

Where M is the mass of the nucleus, m is the electron mass, c is speed of light in vacuum, and E_0 is the electron beam energy. Since $m<<M$, $E_0<<Mc^2$, E_t can rewritten as follows:

$$E_{\max} = \frac{2E_0\left(E_0 + 2mc^2\right)}{Mc^2}$$

Moreover, in our experiment, E_0=200 keV.

Supporting Information References

[1] J. G. McLean, B. Krishnamachari, D. R. Peale, *Phys. Rev. B* **1997**, *55*, 1811.

[2] Z. Horita, *Mater. Trans., JIM* **1998**, *39*, 947.

[3] R. F. Egerton, P. Li, M. Malac, *Micron* **2004**, *35*, 399.

[4] R. F. Egerton, R. McLeod, F. Wang, M. Malac, *Ultramicroscopy* **2010**, *110*, 991.
